# Editorial: Integrative OMICS approaches in cancer biology: from molecular mechanisms to precision medicine

**DOI:** 10.3389/fonc.2026.1960688

**Published:** 2026-09-15

**Authors:** Rajiv Pathak, Vibhav Gautam, Rajiv Kumar, Lalit Kumar

**Affiliations:** 1 Department of Genetics, Albert Einstein College of Medicine, New York, NY, United States; 2 Centre of Experimental Medicine and Surgery, Institute of Medical Sciences, Banaras Hindu University, Varanasi, Uttar Pradesh, India; 3 Department of Urology, Institute of Medical Sciences, Banaras Hindu University, Varanasi, Uttar Pradesh, India

**Keywords:** cancer biology, integrative omics, molecular mechanisms, omics approaches, precision medicine

Despite significant advances in cancer diagnosis and treatment, cancer remains a major cause of disease burden and mortality worldwide (1). Its complexity arises from extensive molecular and cellular heterogeneity, which influences tumor initiation, progression, metastatic spread, and treatment response (2). The rapid evolution of high-throughput OMICS technologies, including genomics, transcriptomics, proteomics, epigenomics, metabolomics, and single-cell profiling, has significantly advanced cancer research by enabling comprehensive characterization of tumors across multiple molecular layers and biological scales (3–5). Integrating these complementary OMICS datasets has improved our understanding of the molecular basis of tumor heterogeneity, enabling the identification of clinically relevant biomarkers, improved tumor classification, and new therapeutic targets to support precision oncology (6–9). The articles included in this Research Topic collectively illustrate how advances in integrative OMICS are transforming cancer research, spanning methodological innovations, molecular profiling, biomarker discovery, liquid biopsy, and therapeutic strategies.

The first contribution to this Research Topic is a mini-review on gene expression normalization, highlighting that the reliability of gene expression-based biomarkers critically depends on rigorous methodological design and robust normalization strategies. Pathak et al. provides a concise and insightful review of the evolution of computational algorithms for reference gene selection in quantitative reverse transcription PCR (qRT-PCR), a widely adopted technique in cancer biology and precision medicine. The authors critically evaluate widely used normalization approaches, including geNorm, NormFinder, BestKeeper, gQuant, and emerging computational strategies, highlighting their respective strengths, limitations, and applications across diverse experimental settings. By emphasizing the importance of selecting stable reference genes for accurate gene expression quantification, this mini review provides a valuable framework for improving the reproducibility, accuracy, and translational relevance of qRT-PCR-based biomarker discovery and molecular diagnostics in precision oncology.

Vargas-Castellanos et al. present a prospective study protocol designed to generate the first integrated germline and transcriptomic dataset for epithelial ovarian cancer (EOC) in a Colombian population. By combining next-generation sequencing of hereditary cancer-associated genes with RNA sequencing of paired peripheral blood and tumor tissue samples from treatment-naïve patients, the study aims to identify population-specific molecular signatures, characterize the tumor microenvironment, and uncover clinically relevant biomarkers associated with EOC. Importantly, this work addresses the underrepresentation of Latin American populations in cancer genomic research and establishes a valuable framework for improving molecular stratification and advancing precision oncology in diverse patient populations.

Highlighting the clinical potential of multimodal liquid biopsy and single-cell technologies in precision oncology, Wolańska et al. present a case report of a patient with stage IVB high-grade serous ovarian cancer (HGSOC) who achieved an exceptionally favorable response to treatment. By integrating single-cell RNA sequencing of peripheral blood mononuclear cells, platelet bulk RNA sequencing, and imaging flow cytometry for circulating tumor cell analysis, the authors identified distinctive immune and metabolic signatures, including upregulation of IL-12 signaling-related genes together with alterations in mTOR signaling, angiogenesis, and glycolytic pathways. Although based on a single patient, this integrative liquid biopsy approach demonstrates how multimodal blood-based profiling can generate patient-specific molecular insights, supporting the development of minimally invasive biomarkers and personalized strategies for ovarian cancer management.

Hakozaki et al. introduce an innovative proteogenomic strategy for the non-invasive monitoring of non-muscle-invasive bladder cancer (NMIBC) by detecting tumor-specific somatic mutant proteins in urinary extracellular vesicles (EVs). By integrating whole-exome sequencing of tumor tissues with deep mass spectrometry-based proteomic profiling, the authors demonstrate that tumor-derived mutant proteins, including LCP1_D321H, TKT_K102N, and PLCD1_R639H, can be detected and quantitatively monitored in urinary EVs. Importantly, the abundance of these mutant proteins closely correlated with cystoscopically confirmed tumor burden, decreased markedly following tumor resection, and remained undetectable during recurrence-free follow-up. This proof-of-concept study establishes urinary EV-based proteogenomics as a highly specific liquid biopsy approach for monitoring bladder cancer recurrence, providing a promising alternative to conventional cystoscopic surveillance and advancing precision oncology through personalized biomarker development.

Beyond nucleic acid- and protein-based molecular profiling, metabolic and endocrine factors are increasingly recognized as important contributors to cancer progression. Raj et al. investigate the clinical significance of serum omentin (ITLN1), an adipokine involved in metabolic homeostasis and inflammatory regulation, as a potential biomarker in breast cancer. The authors demonstrate that elevated circulating omentin levels are associated with adverse clinicopathological features, such as increased tumor size, lymph node metastasis, and advanced disease stage, suggesting a close association with tumor aggressiveness and breast cancer progression. Collectively, these findings highlight the potential of serum omentin as a minimally invasive biomarker associated with breast cancer progression, supporting its utility for improved patient risk stratification and prognostic assessment.

Finally, translating molecular insights into improved therapeutic strategies is addressed in a systematic review and meta-analysis by Ke et al., who synthesized evidence from 41 randomized controlled trials evaluating traditional Chinese medicine (TCM) combined with immune checkpoint inhibitors (ICIs) across multiple malignancies, including lung, liver, gastric, esophageal, colorectal, and ovarian cancers. The authors demonstrate that the combination therapy is associated with improved objective response and disease control rates, prolonged survival outcomes, enhanced immune function, and a reduced incidence of treatment-related adverse reactions compared with ICIs alone. Collectively, this comprehensive analysis highlights the potential of integrative therapeutic strategies to enhance immunotherapy efficacy and improve clinical outcomes in precision oncology.

Taken together, the six contributions in this Research Topic underscore the transformative role of integrating robust molecular data with rigorous analytical approaches in advancing precision oncology. Spanning methodological advances in gene expression analysis, population-specific genomic profiling, proteogenomics, circulating biomarkers, multimodal liquid biopsy, and evidence-based therapeutic strategies, these studies collectively demonstrate how integrative OMICS-based approaches can improve our understanding of cancer biology while accelerating biomarker discovery, improving patient stratification and clinical outcomes. We hope that the work presented in this Research Topic will stimulate further interdisciplinary collaboration among experimental biologists, computational scientists, and clinicians, fostering the development of next-generation OMICS-driven approaches for cancer diagnosis, prognosis, surveillance and personalized cancer therapy.

## References

[1] Bray F Laversanne M Sung H Ferlay J Siegel RL Soerjomataram I . Global cancer statistics 2022: GLOBOCAN estimates of incidence and mortality worldwide for 36 cancers in 185 countries. CA Cancer J Clin. (2024) 74:229–63. doi: 10.3322/caac.21834 38572751

[2] Zhang J Li H Ru S Feng H Guo J Sun D . Tumor heterogeneity: development, mechanisms, and therapeutic implications. Signal Transduct Target Ther. (2026) 11:271. doi: 10.1038/s41392-026-02749-7 42437748 PMC13358051

[3] Hanahan D . Hallmarks of cancer: New dimensions. Cancer Discov. (2022) 12:31–46. doi: 10.1158/2159-8290.cd-21-1059 35022204

[4] Heo YJ Hwa C Lee GH Park JM An JY . Integrative multi-omics approaches in cancer research: From biological networks to clinical subtypes. Mol Cells. (2021) 44:433–43. doi: 10.14348/molcells.2021.0042 34238766 PMC8334347

[5] Menyhart O Gyorffy B . Multi-omics approaches in cancer research with applications in tumor subtyping, prognosis, and diagnosis. Comput Struct Biotechnol J. (2021) 19:949–60. doi: 10.1016/j.csbj.2021.01.009 PMC7868685 33613862

[6] Tomar S Siddiqui S Pathak R Srivastava V . Unlocking the potential of immunomodulators as synergistic immune-based therapies in cancer. Discov Med. (2025) 37:411–32. doi: 10.24976/discov.med.202537194.35 40116091

[7] Rajneesh Tiwari R Singh VK Kumar A Mehrotra S Gautam V . Exploring metabolic and immunological biomarkers for oral squamous cell carcinoma: Potential targets for precision therapy. Biol (Basel). (2025) 14:1109. doi: 10.3390/biology14091109 41007256 PMC12467829

[8] Kumari V Tiwari H Singh S Kailashiya V Hoai NTT Sachan S . AI-driven multi-omics integrated applications using diverse neural networks for breast cancer diagnostic screening and biomarker discovery. Med Res Rev. (2026). doi: 10.1002/med.70078 42489168

[9] Tiwari H Singh S Sharma S Gupta P Verma A Chattopadhaya A . Deciphering the landscape of triple negative breast cancer from microenvironment dynamics and molecular insights to biomarker analysis and therapeutic modalities. Med Res Rev. (2025) 45:817–41. doi: 10.1002/med.22090 39445844

